# Influence of neurovascular anatomy on perforation site in different mouse strains using the filament perforation model for induction of subarachnoid hemorrhage

**DOI:** 10.1371/journal.pone.0263983

**Published:** 2022-10-13

**Authors:** Vanessa Weyer, Máté E. Maros, Stefanie Kirschner, Samantha Krost-Reuhl, Christoph Groden, Martin Kramer, Marc A. Brockmann, Andrea Kronfeld

**Affiliations:** 1 Department of Neuroradiology, University Medical Center Mainz, Mainz, Germany; 2 Medical Faculty Mannheim, Department of Neuroradiology, University of Heidelberg, Mannheim, Germany; 3 Medical Faculty Mannheim, Department of Radiation Oncology, University of Heidelberg, Mannheim, Germany; 4 Department of Veterinary Clinical Sciences, Small Animal Clinic, Justus-Liebig-University Giessen, Giessen, Germany; Henry Ford Health System, UNITED STATES

## Abstract

**Background:**

Filament perforation is a widely-used method to induce subarachnoid hemorrhage (SAH) in mice. Whereas the perforation site has been assumed to be in the branching of middle cerebral artery (MCA) and anterior cerebral artery (ACA), we recently observed more proximal perforations.

**Methods:**

Filament perforation was performed in CD1- (n = 10) and C57Bl/6N-mice (n = 9) ex vivo. The filament was left in place and the perforation site was microscopically assessed. Digital subtraction angiography (DSA) was performed in CD1- (n = 9) and C57Bl/6J-mice (n = 29) and anatomical differences of the internal carotid artery (ICA) were determined.

**Results:**

Whereas in C57Bl/6N-mice perforation occurred in the proximal intracranial ICA in 89% (n = 8), in CD1-mice the perforation site was in the proximal ICA in 50% (n = 5), in the branching between MCA and ACA in 40% (n = 4), and in the proximal ACA in 10% (n = 1). DSA revealed a stronger angulation (p<0.001) of the ICA in CD1-mice (163.5±2.81°) compared to C57Bl/6J-mice (124.5±5.49°). Body weight and ICA-angle showed no significant correlation in C57Bl/6J- (r = -0.06, p_weight/angle_ = 0.757) and CD1-mice (r = -0.468, p_weight/angle_ = 0.242).

**Conclusion:**

Filament perforation in mice occurs not only at the hitherto presumed branching between MCA and ACA, but seems to depend on mouse strain and anatomy as the proximal intracranial ICA may also be perforated frequently.

## Introduction

Subarachnoid hemorrhage due to aneurysm rupture is associated with a high morbidity and mortality (over 30% within the first 24 hours) [1,2] and more than 30% of the surviving patients suffer from delayed cerebral ischemia [3]. Due to the lack of sufficient therapeutic regimens, animal experiments are still being carried out to solve these problems.

The mouse is a widely used model for SAH as it resembles the pathophysiology in humans closely [4] and the number of publications describing different models to induce SAH has been increasing within the past decades [5]. Besides single or repeated injection of blood into the cisterna magna or the prechiasmatic cistern and intracisternal vessel transection, the filament perforation model is the most frequently used method in mice [6]. The main reason for the extensive use of this technique has been discussed to be the fact, that it mimics the situation of cerebral aneurysm rupture most closely [7].

Whereas some papers provide a step-by-step description of the surgical procedure for filament perforation [6], information regarding the exact site of vessel perforation is sparse and remained a controversial issue [5]. With several papers schematically illustrating the perforation site to be located in the “carotid T” (i.e. the branching of middle cerebral artery (MCA) and anterior cerebral artery (ACA)) [5,8,9], this fact often has been taken for granted. In one of our last studies, however, we observed growth of pseudoaneurysms in live mice using digital subtraction angiography (DSA) in the proximal ICA following filament perforation [10], which raised our attention and made us study the exact site of vessel perforation in the murine filament model more closely. This led us to the question to which extent anatomical differences and even the use of a specific mouse strain may be responsible for this finding.

In the underlying study we performed filament perforation in two different mouse strains (CD1 and C57BL/6) and afterwards analyzed the site of perforation microscopically. Additionally, we performed measurements of the angulation of the intracranial ICA right after leaving the skull base, where we observed to be another frequent site of perforation.

## Materials and methods

All experiments were carried out after receiving approval by the local governmental committee (Regierungspräsidium Karlsruhe, Germany; approval number: 35–9185.81/G-42/16). Institutional guidelines for animal welfare and experimental conduct were followed and the experiments have been reported in compliance with the ARRIVE guidelines.

### Filament perforation (ex vivo)

Filament perforation was performed in nineteen female eight to fourteen week-old mice (n_CD1_ = 10 weighing 35-44g and n_C57Bl/6_ = 9 weighing 19-23g). The filament perforation model was performed as described below (and by other groups [4,10,11]), except that the animals in this study group were euthanized right after anesthesia and before the surgery by an intraperitoneal injection of a mixture of ketamine (120mg/kg) and xylazine (16mg/kg), and finally euthanized by an intracardial injection of the same mixture. Thus, filament perforation was performed just after determination of death. Briefly, the external, internal and common carotid artery were exposed and the external carotid artery (ECA) was traced in the direction of the digastric muscle, ligated and cut. The pterygopalatine artery (PPA) was exposed and ligated. An arteriotomy was performed on the distal part of the ECA stump. A stiff and blunted monofilament thread (6–0 in C57Bl/6 and 5–0 in CD1) (Prolene, Ethicon Johnson & Johnson, Belgium, EU) was inserted through the arteriotomy into the ECA and pushed forward through the ICA until a slight resistance was felt. At this point, the filament was pushed approx. 2 mm further to perforate the vessel wall. The filament was not withdrawn after the perforation, but remained in the vessel to subsequently identify the perforation site.

**Fig 1:**
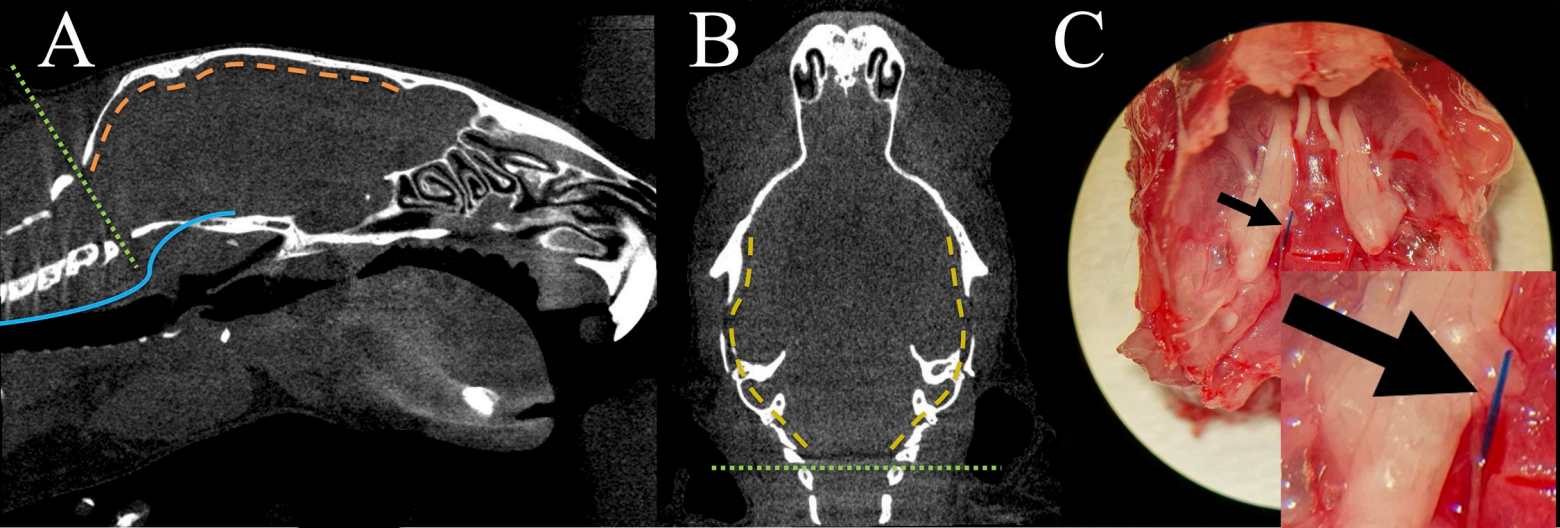
Sagittal (**A**) and transversal (**B**) reconstruction of a micro-CT image from a C57Bl/6-mouse. Because the filament used for perforation (**A**, *blue line*) runs at the skull base, the mouse was decapitated with a scissor along the *green dotted line* (**A, B**). Beginning at the foramen magnum the skull was opened with a blunt forceps by cracking it laterally on both sides (**B**, *yellow dashed line*) and finally the whole calvarium was removed (**A,** *orange dashed line*). After careful removal of the brain, the filament can be spotted at the site of perforation outside the vessel (**C, black arrow**).

To identify the perforation site, surgical preparation of the skull base was carried out by cutting around the surgical area leaving the filament exactly in position. Afterwards, skin and fur were removed from the skull. Subsequently, remaining cervical vertebrae were removed to access the brain (Fig 1A and 1B; green dotted line). An atraumatic and blunt forceps was used to open the skull by breaking the skullcap laterally starting at the foramen magnum (Fig 1B; yellow dashed lines) and finally the whole calvarium was removed (Fig 1A; orange dashed line). Subsequently the head was turned around. By applying slight pressure with a forceps on the cerebellum, a small gap between the brain and the skull base was opened. From here the filament inside the ICA could be tracked from the carotid channel, through which the ICA runs, and afterwards the intracranial part of the ICA. Finally, the filament was cut with a small Vannas scissor and the brain was taken out of the skull by dissecting the optic nerve and the olfactory bulb (Fig 1C).

### Intravenous digital subtraction angiography (in vivo)

DSA data from thirty-eight female eight to fourteen week-old mice (n_CD1_ = 9 weighing 27-42g and n_C57Bl/6_ = 29 weighing 18-24g) were used. Intravenous DSA was performed using an industrial micro-CT (Y.Fox; Yxlon, Garbsen, Germany) equipped with an open multifocus x-ray tube and a 14-bit silicon flat panel detector (Varian PaxScan 2520 D/CL; Varian, Palo Alto, CA, USA). For contrast agent applications a vascular access mini port (VAMP) was custom-made as previously described [12] and implanted into the right jugular vein [10]. Briefly, the animals were anesthetized by subcutaneous injection of a mixture of medetomidine (0.5 mg/kg), midazolam (5 mg/kg) and fentanyl (0.05 mg/kg) (MMF). When sufficient anesthesia depth was achieved, mice were positioned in a mouse cradle [13], the field of view has been set and a gain-and-offset calibration was started. Intravenous DSA was performed by infusion of 250 μl of pre-warmed (37°C) contrast agent bolus (Iomeprol 400; infusion rate 3.4 ml/min) via the VAMP, which was attached to a 1 ml syringe by a catheter. The syringe was pre-filled with contrast agent and placed into an infusion pump (PHD 2000; Harvard Apparatus, March-Hugsetten, Germany). Images were acquired at 30 frames per second, using a tube voltage of 80 kV and 75 μA current. Acquisition time was manually determined starting a few seconds before contrast agent application. Spatial resolution ranged between 13x13μm and 16x16μm. Intravenous DSA sequences were recorded in an Audio Video Interleave (AVI) movie file format.

Two days after the initial i.v. DSA, these mice were induced a SAH in-vivo by using the filament perforation model [10]. The animals were anesthetized (MMF) and after the filament perforation model was performed as described above. However, after perforating the vessel, the filament was retracted into the ECA stump, thereby allowing full reperfusion of cerebral vessels via the ICA and inducing SAH. Finally, the filament was withdrawn from the ECA and the ECA was ligated. The wound was closed with a simple interrupted suture (6–0 Vicryl, Ehticon, Norderstedt). Postoperatively all animals were antagonized by subcutaneous injection of a mixture of atipamezole (2.5 mg/kg), flumazenil (0.5 mg/kg) and naloxone (1.2 mg/kg). All animals underwent i.v. DSA every other day after induction of SAH [10].

### Image processing and statistical analysis

**Fig 2:**
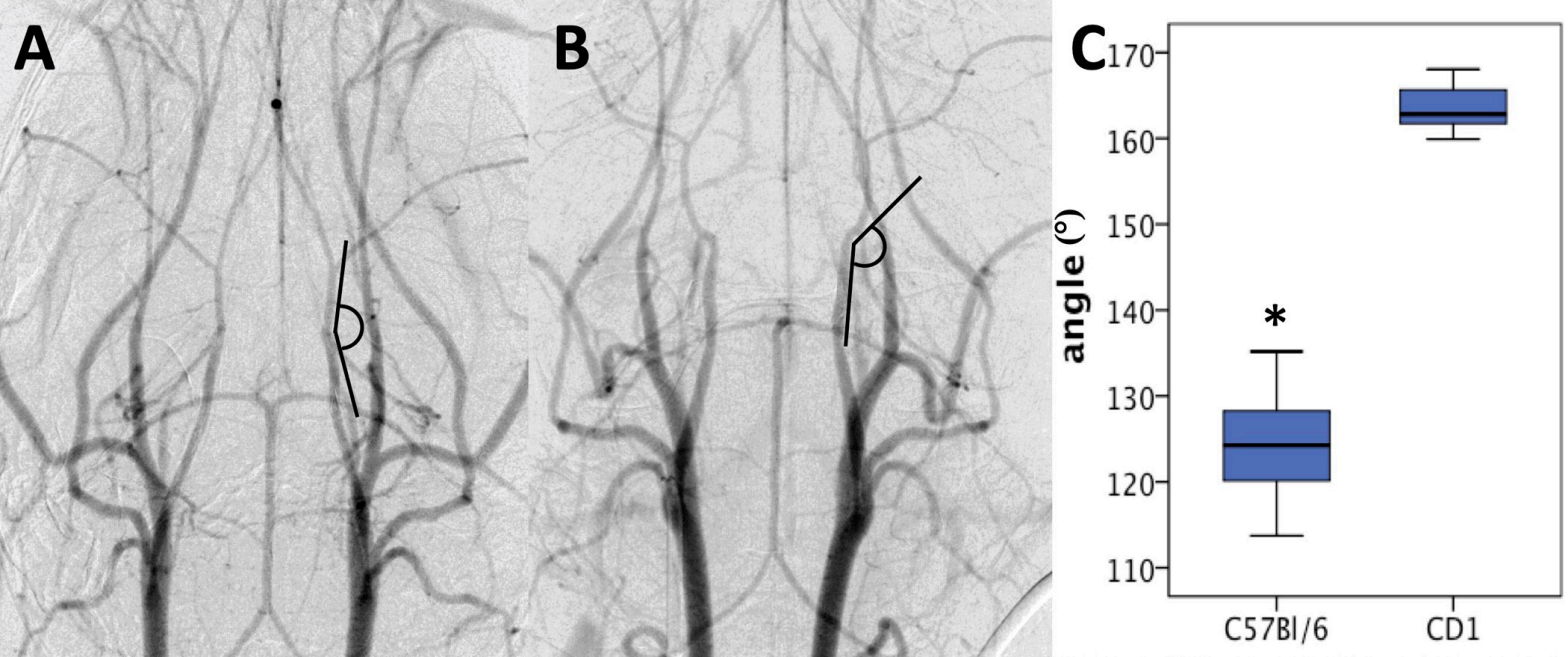
DSA in live mice demonstrates differences in neurovascular anatomy in CD1- and C57Bl/6-mice (A-C). The angle of the proximal intracranial ICA was measured in CD1- (**A**) and C57Bl/6-mice (**B**). Measurements revealed a significant difference of the mean ICA-angle between CD1- (163.54°) and C57Bl/6-mice (124.54°; p<0.001) (**C**).

The public domain software ImageJ (ImageJ 1,47v; Wayne Rasband, National Institutes of Health, USA) was used for postprocessing of the recorded AVI files. Movie files were imported and decomposed into single projections. Six to eight single projections were chosen and overlaid with an opacity of 25% and the angle of the ICA as it enters the neurocranium was measured (Fig 2). To yield comparable results due to variable magnifications, the intravenous port was used to calibrate measurements.

### Statistics

Statistics were conducted with IBM SPSS Statistics for Windows, version 25 (IBM Corp., Armonk, N.Y., USA). Relations between body weight, age and ICA-angle were analyzed with a descriptive statistic using the Pearson correlation coefficient. Measurements are provided as mean ± 1 standard deviation, unless indicated otherwise. Differences of the ICA-angle between the mouse strains were determined by an unpaired t-test. P values <0.05 were considered statistically significant.

## Results

### Surgery time and technical difficulties

In CD1-mice, preparation of vessels to insert the filament took significantly longer compared to C57Bl/6-mice (t_CD1_ = 19.67+/-1.91min; t_C57Bl/6_ = 14.89+/-0.98min; p<0.001), which we attributed to strain-specific higher body weight in CD1-mice. Filament perforations were performed alternating between CD1- and C57Bl6-mice to exclude systematic errors. In three CD1-mice induction of SAH showed to be difficult, because the slight resistance, which should be felt, was missing. In these three animals, however, successful induction of SAH later was confirmed by microscopic examination.

### Microscopic evaluation of the perforation site

In the previously euthanized CD1-mice (n_CD1_ = 10) and C57Bl/6N-mice (n_C57Bl/6N_ = 9) the precise perforation spot of the filament was identified after careful removal of the brain. Fig 2A illustrates the different sites of perforation in relation to the different mouse strains (2A). Briefly, in CD1-mice in five cases (50%) the filament perforated in the proximal part of the intracranial ICA (exemplarily shown in Fig 2D and a closeup in Fig 2E), whereas in four cases (40%) the filament perforated the vessel at the carotid T (exemplarily shown in Fig 2B and a closeup in Fig 2C). In one case (10%) the filament slipped into the ACA, where the vessel was perforated proximally. In C57Bl/6N-mice, the proximal part of the ICA was perforated eight times (88.9%) and only in one case (11.1%) the filament perforated at the carotid T.

Despite previous death and therefore missing blood flow, in 11/19 animals a distinct amount of blood was visible on the brain surface in direct vicinity of the perforation site.

### Relationship between body weight, age, and ICA-angle in C57Bl/6J- and CD1-mice (in-vivo)

The average body weight of nine 12.0±4.0 week-old CD1-mice was significantly higher than that of twenty-nine 11.5±2.1 week-old C57Bl/6J-mice (37.1±5.8g vs. 20.9±1.66g; p<0.001).

**Fig 3:**
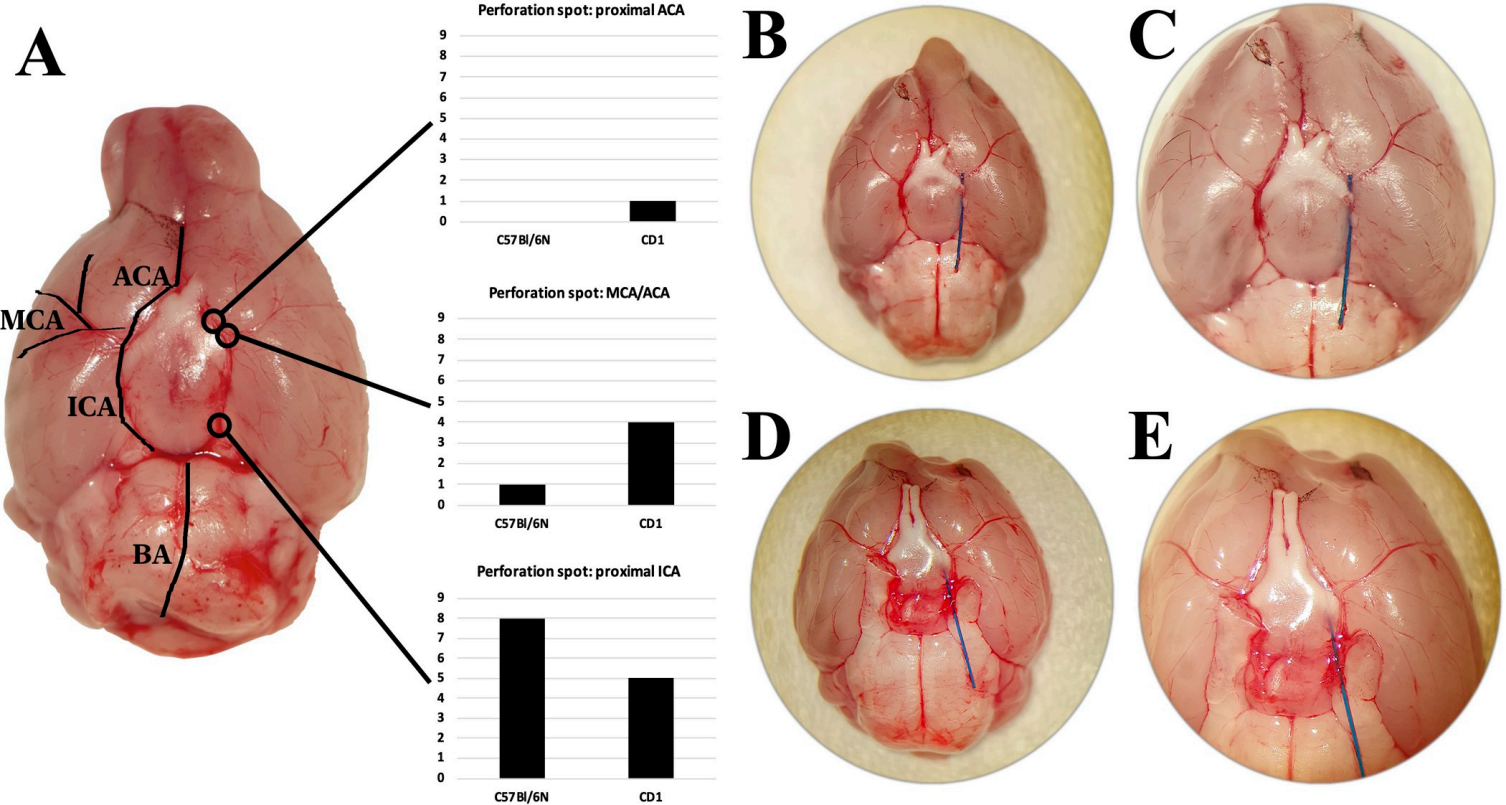
Microscopical evaluation of the perforation spot in CD1- and C57Bl/6-mice (**A**). In C57Bl/6N-mice in most cases the filament perforated the proximal intracranial ICA (n = 8/9; A) (exemplary images; **D** 25x magnification, **E** 40x magnification) and seldomly the distal intracranial ICA (n = 1/9). In CD1-mice the proximal intracranial ICA was perforated in 5/10 mice, the distal ICA in 4/10 mice (exemplary images; **B** 25x magnification, **C** 40x magnification; it is noteworthy to mention that the filament tip ends in parenchymal tissue) and the proximal ACA in 1/10 mice.

Intravenous DSA (Fig 3A–3C) revealed the angle of the proximal intracranial ICA in C57Bl/6J-to be relatively flat (124.5±5.5°) compared to the angle of the proximal intracranial ICA in CD1-mice (163.5±2.8°; p<0.001 between both mouse strains).

In both mouse strains body weight and age showed a highly significant positive correlation (C57Bl/6J: r = 0.527, p_weight/age_<0.01; CD1: r = 0.895, p_weight/age_<0.01). Body weight and angle of the intracranial part of the ICA did not correlate in C57Bl/6J-mice (r_sp_ = -0.06, p_weight/angle_ = 0.757), whereas in CD1-mice we observed a moderate negative, non-significant correlation (r_sp_ = -0.468, p_weight/angle_ = 0.242). In C57Bl/6J-mice ICA angle and age showed a weak positive correlation of no significance (r = 0.24, p_angle/age_ = 0.209). Likewise in CD1-mice a moderate negative correlation did not reach significance (r = -.0.359, p_angle/age_ = 0.382).

### Detection of pseudoaneurysms after filament perforation in living mice

**Fig 4:**
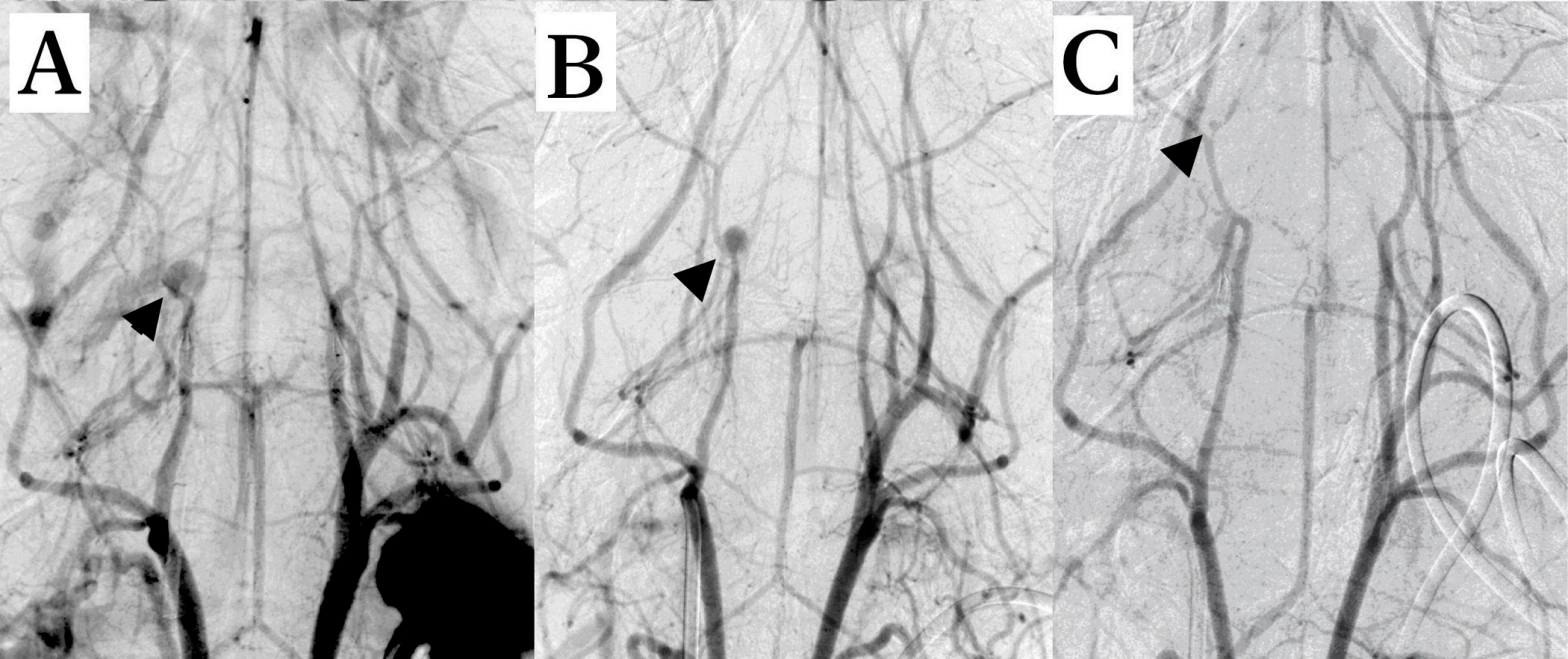
In vivo DSA showed intracranial pseudoaneurysms in three C57Bl/6J mice following filament perforation: Two pseudoaneurysms originated from the proximal intracranial part of the ICA and were detected two days after induction of SAH (**A, B, black arrowheads**). Furthermore, a small aneurysm originating from the proximal ACA was also detectable (**C, black arrowhead**).

In three C57Bl/6-mice pseudoaneurysms of the intracranial arteries were identified in DSA two days after induction of SAH (Fig 4A–4C). In two cases the aneurysm originated from the proximal intracranial ICA (Fig 4A and 4B), whereas in one mouse the structure was linked to the proximal ACA (Fig 4C).

## Discussion

The study of SAH-related cerebral vasospasm and DCI remains challenging. Although using human vessels for ex vivo experiments is feasible [14], this approach by far does not resemble the extremely complex biological interactions following SAH. Likewise, postmortem pathological examinations of human or animal arteries provide only a limited amount of information [15]. Therefore, animal experiments are inevitable to further push our understanding of SAH-related cerebral vasospasm and DCI. Unfortunately, murine models of SAH are technically demanding and may be difficult to standardize [11,16].

In a systematic review by Marbacher et al. [17] the variability of experimental parameters in current models of SAH was discussed. The size of filaments used in the perforation model, for example, ranged between 4–0 and 6–0 and the extent of SAH has been reported to depend on the size of the suture used to perforate the artery [8]. On the other side, in a recent study we observed differences in dissemination and degree of SAH using only one size of filament in a single mouse strain [10].

A hitherto neglected topic is the exact localization of perforation when inducing subarachnoid hemorrhage using a filament. Referring to the literature, the perforation spot has been described to be at the terminal branching of the ICA into the ACA and MCA [5,8,9,18–22]. Likewise, oral presentations dealing with this topic frequently illustrate a filament perforating at this point. A problem here is that in ex vivo examinations of extracted brains after induction of subarachnoid hemorrhage it would be difficult to identify the exact localization of perforation, as the blood disseminates throughout the subarachnoid space and the blood clot overlays the extremely small hole. We therefore performed microscopic examination of brains after immediately after filament perforation in dead mice leaving the filament in place.

Only few authors discuss the site of perforation. Bederson et al. indicated, that in some cases even the ICA could be perforated [23], and Muroi et al. [5] doubted that perforation of the ACA is feasible, although this has been claimed by other investigators [24–26]. Our results confirm that i.) perforation of the ICA happens more frequently than expected an seems to depend on the mouse strain and anatomy of brain vessels, and that ii.) perforation of the ACA is feasible: In one case we found the filament to perforate the proximal ACA of a dead C57Bl/6-mouse, and in another case we detected a very small pseudoaneurysm of the proximal ACA in a living mouse using DSA (Fig 3F).

The influence of the mouse strain on the localization of perforation was not discussed in the literature before. The mouse strains C57Bl/6 and CD1 are the most used strains in context with the filament perforation model [17]. In the underlying study we observed differences in anatomy of the brain vessels, which could not be attributed to age or weight of the animals but seems to affect the localization of filament perforation. This in context with other publications reporting significant anatomical variations for different mouse strains [27]. We found the supraaortic and intracranial vascular structures in CD1 mice to appear elongated compared to C57Bl/6 mice, in which the circle of Willis is more heart-shaped. Thus, in CD1 mice the possibility to push the filament straight forward to the intracranial bifurcation of the ICA seems much higher and the probability to perforate the proximal intracranial ICA seems to be lower in CD1 mice. In C57Bl/6-mice featuring a pronounced angle of the proximal intracranial ICA the probability to perforate the proximal ICA seems to be higher. This is well in line with our results.

In larger CD1-mice we found a thicker 5-0-filament to be helpful to successfully perforate the vessel without being at risk of slipping into smaller vessels (like the ACA). A 5–0 filament, however, may be difficult to use in C57Bl/6-mice due to its diameter being too large for the smaller vessels of this mouse strain. Nevertheless, our data show that using a thicker filament in CD1-mice results in more proximal perforations more frequently. In C57Bl/6-mice using a thinner filament resulted more frequently in more distal perforations. However, we cannot define if this clinical observation is just a coincidence (of perforation site and mouse strain anatomy or of perforation site and filament size), since no controlled experiments investigating the effect of filament size upon perforation site were carried out. Another point which needs to be discussed is the fact, that one cohort of animals was sacrificed directly before filament perforation. Here from a theoretical point of view one cannot completely rule out, that the lack of blood flow in these animals might be influencing the results. On the other hands, since mice were sacrificed directly before the surgery, we do not think that there should be a relevant influence (e.g. increased friction) due to intravascular hemostasis. Furthermore, once a filament is advanced in a living mouse, the blood flow should be significantly reduced as well. One could even speculate, that while the filament is inserted the blood flow is reversed in parts of the intracranial ICA for a short time. This is a phenomenon we frequently see in patients with large vessel occlusion and which we already observed in DSA of mice (unpublished data).

In most studies using the filament perforation model, description of the surgical procedure is similar. Exact information regarding the distance to successfully perforate a vessel, however, is sparse. A slight resistance might be felt when the filament tip reaches the bend of the proximal part of the ICA. But how can the operator know how far further to push a filament? Pushing the filament 3–5 mm further would not only cause a vessel rupture, but could also induce relevant damage of the brain with far-reaching consequences like neurological abnormalities, which could influence the neuroscore and experimental outcome. If not pushed far enough, mice will not develop SAH. Therefore several investigators started to use additional invasive monitoring techniques including intracranial pressure monitoring in mice [7,11] and less invasive techniques like micro-CT have been used to verify the grade of the induced SAH [10]. It thus might be difficult to standardize this technique for different mouse strains as many factors may play a role like stiffness and length of the filament, technique and experience of the investigator, site of the arteriotomy, mouse strain and anatomy, and even the position of the animals’ head during surgery has been discussed to influence filament perforation [5].

On the other hand, it remains unclear whether more proximal or more distal perforation leads to any relevant differences. One could consider i.) an altered neurological state and earlier death of a mouse in case of more proximal perforation with subsequent brain injury due to the filament, b.) the development of pseudoaneurysms that might (re-)rupture during an ongoing study and result in sudden death of an animal, or c.) simply more or less strong SAH (due to smaller or larger perforations or perforation of more proximal or more distal vessels) or diverging distributions of SAH affecting an animals neuroscore and outcome. From previous studies it is known, that the volume of blood produced in this model depends on the suture size used to perforate the artery, and even using the same suture size throughout an experiment the amount of SAH varies [8,28], which we were also able to observe in one of our recent studies using micro-CT to grade SAH after filament perforation [10]. In this recent study we also observed sudden death of two animals, which might has been related to rupture of pseudoaneurysms, as these animals were well before. Unfortunately, we did not perform autopsy in all of these animals in this recent study.

Like humans, mice develop large vessel vasospasm (LVV) following SAH [18–21,29]. LVV in mice, however, is less pronounced and the clinical impact of LVV in mice is much smaller, as mice seldomly develop relevant LVV-related territorial ischemia. Furthermore, the temporal progress of LVV in mice differs from that in humans: in mice LVV peaks between day 2–3 after SAH [10], compared to days 7–10 in humans [30,31]. One of the reasons for this difference might be the lissencephalic structure of the murine brain [6,32,33], which has been discussed to support clearance of the subarachnoid blood, while this process in gyrencephalic primates might take longer. Detailed information about the rate of clot clearance in mice after SAH, however, is hard to find in the literature. In a study using the cisterna magna injection model the authors described that one hour after SAH a large diffuse blood clot was observed in the basal subarachnoid space of the brain; on day 1 the clot was thinner, but still clearly visible around the major cerebral arteries; on days 3 and 4 the cisternal blood clot was hardly to detect [32]. Thus, the distribution of blood in the subarachnoid space might have an impact on the development and duration of vasospasm in mice and therefore the site and size of perforation may influence the outcome stronger than expected.

To conclude, multiple factors such as animal strain, filament size and insertion depth, as well as varying perforation sites must be considered using the filament perforation model. As pointed out in this manuscript, the exact localization of perforation seems to depend on several factors and it should not be taken for granted, that perforation always happens in the distal ICA at the bifurcation into MCA and ACA.

## Supporting information

S1 File Data—ex vivo. (XLSX)

S2 File Kopie von Statistik C57-CD1 in vivo. (XLSX)

## Abbreviations

ACA: anterior carotid artery
CCA: common carotid artery
DSA: digital subtraction angiography
ECA: external carotid artery
ICA: internal carotid artery
LVV: large vessel vasospasm
MCA: middle cerebral artery
PPA: pterygopalatine artery
SAH: subarachnoid hemorrhage
